# Whole genome sequencing to study antimicrobial resistance and RTX virulence genes in equine *Actinobacillus* isolates

**DOI:** 10.1186/s13567-023-01160-2

**Published:** 2023-04-05

**Authors:** Nick Vereecke, Arlette Vandekerckhove, Sebastiaan Theuns, Freddy Haesebrouck, Filip Boyen

**Affiliations:** 1 Department of Translational Physiology, Infectiology and Public Health, Faculty of Veterinary Medicine, Ghent University, Salisburylaan 133, 9820 Merelbeke, Belgium; 2 PathoSense BV, Lier, Belgium; 3 Department of Pathobiology, Pharmacology and Zoological Medicine, Faculty of Veterinary Medicine, Ghent University, Salisburylaan 133, 9820 Merelbeke, Belgium

**Keywords:** SNP-tree, virulence, microbial genomics, nanopore sequencing, *Actinobacillus* genomospecies 1

## Abstract

*Actinobacillus equuli* is mostly associated with disease in horses and is most widely known as the causative agent of sleepy foal disease. Even though existing phenotypic tools such as biochemical tests, 16S rRNA gene sequencing, and Matrix Assisted Laser Desorption Ionization Time of Flight Mass Spectrometry (MALDI-TOF MS) can be used to identify members of the *Actinobacillus* genus, these methods struggle to differentiate between certain species and do not allow strain, virulence, and antimicrobial susceptibility typing. Hence, we performed in-depth analysis of 24 equine *Actinobacillus* isolates using phenotypic identification and susceptibility testing on the one hand, and long-read nanopore whole genome sequencing on the other hand. This allowed to address strain divergence down to the whole genome single nucleotide polymorphism (SNP) level. While lowest resolution was observed for 16S rRNA gene classification, a new multi-locus sequence typing (MLST) scheme allowed proper classification up to the species level. Nevertheless, a SNP-level analysis was required to distinguish *A. equuli* subspecies *equuli* and *haemolyticus*. Our data provided first WGS data on *Actinobacillus* genomospecies 1, *Actinobacillus g*enomospecies 2, and *A. arthritidis*, which allowed the identification of a new *Actinobacillus* genomospecies 1 field isolate. Also, in-depth characterization of RTX virulence genes provided information on the distribution, completeness, and potential complementary nature of the RTX gene operons within the *Actinobacillus* genus. Even though overall low prevalence of acquired resistance was observed, two plasmids were identified conferring resistance to penicillin-ampicillin-amoxicillin and chloramphenicol in one *A. equuli* strain. In conclusion our data delivered new insights in the use of long-read WGS in high resolution identification, virulence gene typing, and antimicrobial resistance (AMR) of equine *Actinobacillus* species.

**Supplementary Information:**

The online version contains supplementary material available at 10.1186/s13567-023-01160-2.

## Introduction

*Actinobacillus* species are Gram-negative bacteria, causing different diseases in several animals, including swine, cattle, and horses [1]. Even though *Actinobacillus equuli* (*A. equuli*) is most frequently associated with disease in horses, also other *Actinobacillus* species, such as *A. suis* [2], *A. arthritidis* [3], *Actinobacillus* genomospecies 1 [3–5] and *Actinobacillus* genomospecies 2 [3, 6] have been occasionally described in equine samples. Nomenclature changes and the fact that it is very difficult to differentiate some of these species with standard biochemical tests, 16S rRNA gene sequencing, and/or MALDI-TOF MS, make it hard to interpret final identification in previously and currently published reports. *Actinobacillus equuli* is an important cause of septicemia in foals (sleepy foal disease) and is inflicted as primary or secondary pathogen in lower respiratory tract diseases, peritonitis, and meningitis in adult horses [1, 7, 8]. Also, it has been occasionally linked with infections in pigs, rabbits, and humans [1, 9–12]. The *A. equuli* species contains 2 subspecies, i.e. *A. equuli* subspecies *equuli* and *A. equuli* subsp. *haemolyticus* [13]. Both subspecies can cause disease in horses, even though *A. equuli* subsp. *haemolyticus* has been associated more with severe respiratory disease. This is thought to be because of the *A. equuli* toxin (Aqx), a member of the repeats-in toxin (RTX) family, which is encoded by the *aqx* genes in *A. equuli* subsp. *haemolyticus* strains only and contributes to the haemolytic characteristic of this subspecies [14]. *A. equuli* infections are feared by both horse owners and veterinarians due to the high mortality rate of such infections.

*Actinobacillus equuli* affected neonatal foals should be treated aggressively with antimicrobial therapy and supportive care. Both in foals and adult horses, various antimicrobial agents are used for treatment, but beta-lactam antibiotics, often combined with aminoglycosides are most frequently applied. Even though there are some reports on antimicrobial resistance in *A. equuli* [15], antimicrobial susceptibility data are still scarce or limited to specific cases or antimicrobial agents [7, 16, 17], while there seem to be no published data on genetic determinants at all [18].

To study bacterial antimicrobial resistance (AMR) and virulence, whole genome sequencing (WGS) has become affordable and of great interest in the field of microbiology. As exemplified for many other bacterial species in both human and veterinary medicine, the use of WGS data allows to study strain relatedness via phylogenetic inference, to perform virulence gene typing, and identify AMR associated mediators [19–25]. Many of these genes are located on mobile genetic elements (MGEs), such as plasmids, integrons, and transposons [26–28]. These MGEs are considered one of the most important drivers in the dissemination of AMR within and cross species and/or genus level. Within and across the genus of *Actinobacillus*, various plasmids have previously been described as important mediators of AMR [29, 30], but data are largely absent for equine species. The availability of new sequencing methodologies, such as third generation long-read nanopore sequencing (Oxford Nanopore Technologies (ONT)), allowed to use this technique in the generation of complete circular and accurate bacterial whole genomes and plasmids. Therefore, the goal of current study was to evaluate the overall genomic relatedness, a new MLST scheme, and distribution of RTX genes within the genus *Actinobacillus *sensu stricto, along with determining the in vitro susceptibility of well-identified recent equine *Actinobacillus* isolates in relation to genetic resistance mechanisms.

## Materials and methods

### *Actinobacillus* isolates and reference/type strains

Twenty-four *Actinobacillus* isolates were obtained from independent clinically affected horses during the period of 2008–2017, except for strains 3873 and 3874 that were isolated from the same animal on different occasions. The isolates were presumptively identified as *A. equuli* by colony morphology and standard biochemical methods, including haemolysis on sheep blood agar [31]. Identification was also obtained with MALDI-TOF MS (Bruker Daltonik GmbH, Bremen, Germany) using the direct transfer method and α‐cyano‐4‐hydroxycinnamic acid as matrix, according to manufacturer’s guidelines and compared with the Bruker Daltonik database containing 8468 mean spectra projections. We considered identifications with a log score value > 2.0 to be reliable at the species level. Considering that equine *Actinobacillus* species may not be easily distinguished, even with MALDI-TOF MS or 16S rRNA gene sequencing [32, 33], further identification based on WGS data was performed, as described below. Pure cultures of all isolates were stored at −80 °C for further analysis. For WGS, *A. equuli* subsp. *equuli* type strain CCUG 2041^T^ was included as an internal control for long-read sequencing quality validation, as genomic data of this strain have been published before and are available at NCBI (accession CP007715.1). Additionally, *A. equuli* subsp. *haemolyticus* type strain CCUG 19799^T^, *A. arthritidis* type strain CCUG 24862^T^, *Actinobacillus* genomospecies 1 reference strains CCUG 22229 and CCUG 22231, and *Actinobacillus* genomospecies 2 reference strain CCUG 15571 [3], were subjected to WGS to assist in the whole genome phylogenetic inference of *Actinobacillus* species, as no or scarce genomic data for these species were available at the time this manuscript was prepared.

### Extraction of high molecular weight DNA and long-read whole genome sequencing

All clinical *Actinobacillus* strains (*n* = 24) and CCUG type/reference strains (*n* = 6) were grown and subjected to DNA extraction. All colonies from a fresh overnight culture were resuspended in 250 μL dPBS (Gibco) and subjected to high-molecular weight (HMW) DNA isolation using the ZymoBIOMICS DNA MiniPrep Kit (Zymo Research), following manufacturers’ instructions. Resulting HMW DNA was subjected to quantification and quality assessment on a NanoDrop Spectrophotometer after which samples with lowered quality (A_260_/A_230_ < 1.7) were cleaned using CleanNGS (CleanNA) magnetic beads. High-quality HMW DNA was used in a rapid long-read sequencing library preparation (SQK-RBK-004; ONT) using 400 ng input per sample and multiplexing up to 12 samples on a R9.4.1 flow cell (ONT). All samples were sequenced for 48 h, and data were collected as raw fast5 files in the MinKNOW software (ONT). All data were basecalled using the Bonito R&D basecaller (v0.4.0; ONT) with the dna_r9.4.1@v3.3 model on the Ghent University HPC Tier 2 GPU cluster Joltik using 1× GPU (NVIDIA Volta V100 32 GB). Draft genomes were assembled using canu (v2.0 [34]), followed by read mapping and polishing using minimap2 (v2.20 [35]) and medaka (v1.5.0; ONT), respectively. Completeness and accuracy of final consensus genomes was assessed using Kraken2 [36], ribosomal multi-locus sequence typing (rMLST) [37], QUAST (v5.0.2. [38]), and CheckM (v1.1.0 [39]). Genome completeness was evaluated against the 589 *Actinobacillus* spp. marker sets (18 genomes with 1004 marker genes). When all 1004 marker genes were identified, a completeness of 100% was reported. A genome QC report and associated NCBI accession numbers can be found in Additional files 1 and 2.

### Phylogenetic analysis of *Actinobacillus* species

Complete and high-quality *Actinobacillus* species genomes were used in a whole genome single nucleotide polymorphism (SNP)-based analysis using csi phylogeny [40] including all available unique whole genomes of *Actinobacillus* spp. from NCBI (*n* = 72 accessed on 29/03/2022). Due to high divergence within the *Actinobacillus* genus, a subset of these genomes (*n* = 53) was kept for downstream analysis, excluding *A. minor*, *A. indolicus*, “*A. porcitonsillarum*”, *A. succinogenes*, *A. porcinus*, *A. delphinicola*, *A. seminis*, and *A. sp.* GY-402, along with genome duplicates (*n* = 10). Thus, a focus will be maintained on *Actinobacillus *sensu stricto. The *A. equuli* subsp. *equuli* strain CCUG 2041^T^ (CP007715.1) was used as reference strain in both analyses. Also, sequences from the 16S rRNA gene were extracted. Since no MLST scheme is currently available for any *Actinobacillus* species, potential MLST genes (*adk* (adenylate kinase), *atpG* (ATP FOF1 synthase subunit gamma), *deoD* (purine nucleoside phosphorylase), *zwf* (glucose-6-phosphate dehydrogenase), *recA* (recombinase A), *mdh* (malate dehydrogenase), and *pgi* (glucose-6-phosphate isomerase)) were extracted from the genomes based on existing MLST schemes from other *Pasteurellaceae* members [41–45]. An overview of the generation of our new MLST scheme is given in Additional file 3. The new *Actinobacillus* spp. MLST scheme will be hosted on pubmlst.org [46]. Subsequently, the aligned SNPs (csi phylogeny output), 16S rRNA gene allele 1, and concatenated MLST genes were used in maximum likelihood (ML) phylogenetic analyses, using IQ-TREE with the GTR + I + R substitution model and 1000 ultrafast bootstraps (-bb) (v1.6.1; [47]).

### Antimicrobial susceptibility testing

Antimicrobial susceptibility testing was performed using the agar dilution assay [48], using Mueller Hinton agar supplemented with 5% defibrinated sheep blood, since preliminary testing showed that growth in cation–adjusted Mueller Hinton broth was difficult to interpret. An overview of the tested antimicrobials can be found in Table 1. Plates were incubated at 35 °C (± 2 °C) for 20–24 h in 5% CO_2_ enriched atmosphere, due to the fastidious and capnophilic nature of the *Actinobacillus* genus in general. *Staphylococcus aureus* ATCC 29213 and *Escherichia coli* ATCC 25922 were used as quality control strains for all antibiotics tested, while for amoxicillin-clavulanic acid testing additionally *Escherichia coli* ATCC 35218 was used. Since no wild type cut-off values are available for *A. equuli* (EUCAST, 2022), the ECOFF was determined using the “Normalized Resistance Interpretation (NRI)” method (Bioscand AB, Täby, Sweden) [49, 50], following manufacturers’ instructions. When the standard deviation of the normal distribution of wild type MIC values exceeds 1.2 log_2_, the outcome can only result in a tentative estimate of the ECOFF and one can only speak of the “putative wild type group”.

**Table 1:**
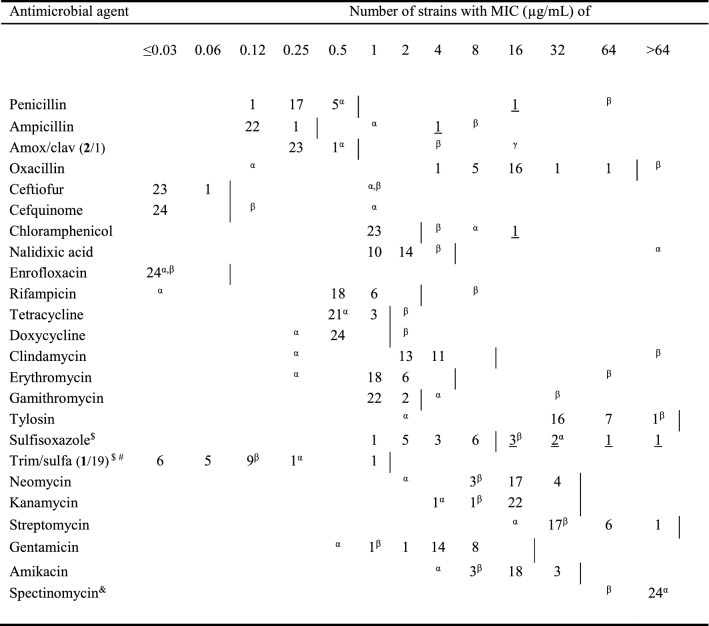
**Distribution of minimum inhibitory concentrations (MIC) of various antimicrobial agents against equine** ***Actinobacillus equuli*** **isolates using the agar dilution test**

The ECOFF, as determined by the NRI method is indicated as a vertical line. The strains showing acquired resistance are underlined. ^#^ the standard deviation of the normal distribution of wild type MIC values exceeds 1.2 log_2_, the outcome can only result in a tentative estimate of the ECOFF and one can only speak of the “putative wild type group”.

^$^ data available for 22 isolates, ^&^ no ECOFF is proposed for spectinomycin because all results were at the upper limit of the tested range; ^α^ indicates result for *S. aureus* ATCC 29,213; ^β^ indicates result for *E. coli* ATCC 25,922; ^γ^ indicates result for *E. coli* ATCC 35,218; for amoxicillin + clavulanic acid (2/1) and trimethoprim + sulfamethoxazole (1/19), the results of amoxicillin and trimethoprim, respectively, are shown.

### Evaluation of molecular mechanisms of virulence and antimicrobial resistance

Virulence and AMR genes were identified with abricate (v1.0.1) [51] using the virulence factor database (VFDB) [52], the comprehensive AMR database (CARD) [53], and a novel RTX toxin database (*n* = 1389), which was composed of RTX toxin and RTX toxin-related protein sequences obtained from NCBI (accessed on 21/02/2023) limited to the family of *Pasteurellaceae* and removal of sequences that were classified as “partial”. Sequences with a sequence homology of at least 60% amino acid identity and 80% coverage were searched for and reported. A complete overview can be found in Additional file 4. In addition, all genomes were annotated using prokka (v1.14.6; [54]), after which all hemolysin-associated genes were extracted and verified in BLASTX (v2.13.0 +). Next to genomes, also plasmids were searched for, analyzed, and manually annotated using open reading frame (ORF) finder (NCBI) and an experimental nr_clustered_blast (NCBI) with default settings. Classification and mobilization potential of identified plasmids was done using the mob-suite (v3.1.0; [55]). All clinical strains were tested for the presence of two identified plasmids using PCR. Based on the obtained plasmid sequences, two primer sets (available in Additional file 5) were generated and used for each plasmid in a *OneTaq* PCR reaction using following thermocycler conditions: initial denaturation at 94 °C for 30 s, 30 cycles of 94 °C for 30 s, 59 °C for 30 s, and 68 °C for 2 min, followed by a final extension at 68 °C for 5 min, using the strain in which the plasmids were originally detected as positive control. After amplification, DNA was visualized on a 1.5% agarose gel and sequenced as described above.

## Results

### Identification and phylogenetic analysis of ***Actinobacillus*** species

All clinical isolates were identified as either *A. equuli* or *A. suis* using MALDI-TOF MS with score values > 2.00, except for one isolate. First and second-best score were often not of the same species, and score values > 2.00 for both *A. equuli* and *A. suis* were frequently observed within one strain. Isolate 3216 showed the best match with *A. pleuropneumoniae* (score value = 2.03) in the MALDI-TOF database and a second best hit with *A. lignieresii* (score value = 1.90). From the WGS data, all ribosomal gene sequences were used to perform a rMLST species identification (pubMLST) which identified all isolate species as *A. equuli* (20–90%), except for isolate 3216 that showed highest support (60%) with *A. lignieresii* (Additional file 2).

**Figure 1:**
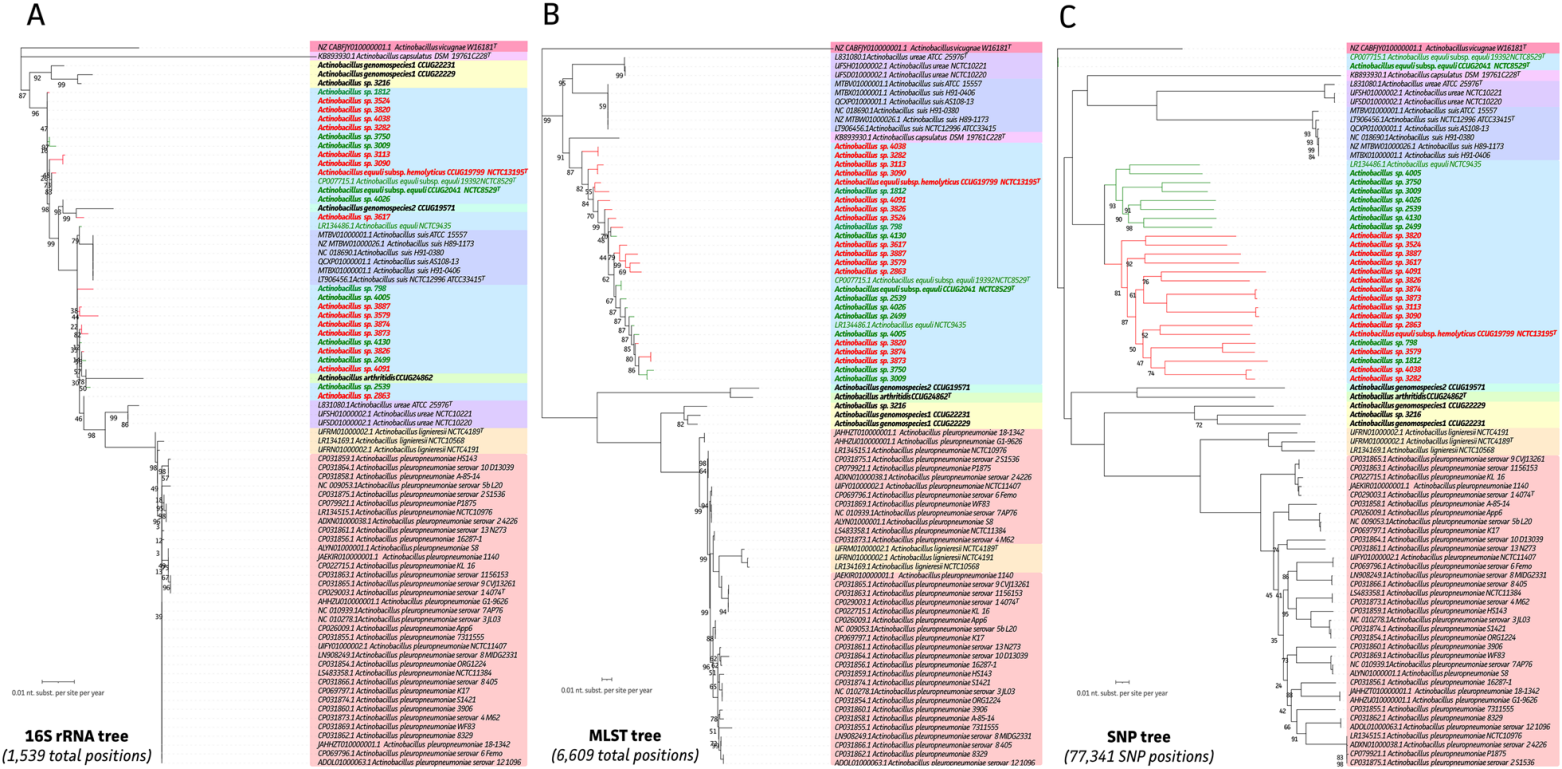
**Maximum likelihood (ML) phylogenetic tree of the *Actinobacillus* genus.** ML trees (1000 ultrafast bootstraps) representing phylogenetic relationship of *Actinobacillus* species on **A** 16S rRNA gene level including allele 1 of each *Actinobacillus* genome; **B** putative MLST level with concatenated *adk*, *atpG*, *deoD*, *zwf*, *recA*, *mdh*, and *pgi* genes; **C** whole genome SNP level (77 341 SNP positions) as compared to the *A. equuli* subsp. *equuli* strain CCUG 2041^T^ (CP007715.1) as reference. Species are highlighted in same colours across different trees, with haemolytic and non-haemolytic *A. equuli* strains highlighted in red and green text, respectively. Tree scales represent number of nucleotide substitutions per site per year.

First, sequencing accuracy of the long-read only assembly was assessed by comparing the newly obtained genome with the available CCUG 2041^T^ (CP007715.1) genomes, showing a 99.999% genome accuracy (Q_50_). A mean genome completeness of 98.7% (± 3.2%) was obtained for the genomes. This is in line with completeness statistics of all available NCBI WGS data (98.7% (± 3.8%)). Hence, the currently obtained genomic data represent highly accurate and complete genomes. To assess the potential use of the 16S rRNA gene and putative MLST scheme for identification and classification of *Actinobacillus* species, specific sequences were extracted from the genomes and used for phylogenetic inferences (Figures 1A and B). Our data was also supplemented with all available *Actinobacillus* spp. genomes (*n* = 62 after duplicate removal). Importantly, eight *Actinobacillus* species showed low whole genome nucleotide overlap with the *A. equuli* reference. These included “*A. porcitonsillarum*” (13%), *A. minor* (12%), *A. indolicus* (8%), *A. porcinus* (5%), *A. seminis* (3%) *A. succinogenes* (3%), *Actinobacillus* sp. GY-402 (3%), and *A. delphinicola* (2%) (Additional file 1). Due to this high genetic divergence and the significant drop in genomic overlap, these species were excluded from further analyses, which limits our main analysis to the genus *Actinobacillus *sensu stricto.

As shown in Figure 1A, the 16S rRNA gene sequence tree does not allow proper distinction of the *A. equuli* species from *A. suis* (purple), *A. arthritidis* (light green), and *Actinobacillus* genomospecies 2 (dark green). While all other species clearly clustered within a separate clade, little 16S rRNA gene specific mutations contributed to the divergence of the *A. lignieresii* and *A. pleuropneumoniae* clades, preventing proper classification. On the other side, our new MLST genes *adk*, *atpG*, *deoD*, *zwf*, *recA*, *mdh*, and *pgi* were concatenated, which allowed proper distinction of all *Actinobacillus* species. The strain 3216 clustered together with the *Actinobacillus* genomospecies 1 strains in both 16S rRNA gene and MLST trees. While this new MLST-based tree allowed proper clustering of all species included in this study, no subspecies resolution for *A. equuli* subsp. *equuli* and *A. equuli* subsp. *haemolyticus* could be obtained. Based on the MLST scheme, the *A. equuli* reference strain CCUG 2041^T^ (CP007715.1) clustered within the *A. equuli* clade. The highest classification resolution could be obtained with the SNP-based WGS phylogenetic analysis (Figure 1C). This allowed to classify all strains, except strain 3216, to the *A. equuli* species with the highest accuracy and resolution. Within the *A. equuli* species, clear distinction could be made between 2 clades. The first clade contained seven non-haemolytic isolates (strains in green) and previously published genomic data of the (non-haemolytic) *A. equuli* reference strain NCTC 9435 (LR134486). The second clade contained 14 haemolytic (strains in red) and two non-haemolytic isolates and previously published genomic data of the *A. equuli* subsp*. haemolyticus* reference strain CCUG 19799^T^. Remarkably, even though both previously published genomes and the currently obtained genome of reference strain CCUG 2041^T^ (CP007715.1) clustered closely together, this cluster seemed to be only distantly related to both current clinical isolates and other *Actinobacillus equuli* reference strains NCTC 9435 and CCUG 19799^T^. As summarized in Additional file 1, the reference strain shared 91.8% (± 0.8%) and 86.4% (± 0.7%) of its genome with the strains belonging to *A. equuli* subsp*. equuli* and *A. equuli* subsp. *haemolyticus*, respectively. Interestingly, based on the MLST analysis, the clade of the *A. lignieresii* species (orange) was wrongly placed within the *A. pleuropneumoniae* clade when compared to the SNP-based tree (Figure 1C). Hence, the SNP-based tree provided the highest resolution of classification and identification for all *Actinobacillus* species. Noteworthy, as seen in the horizontal distances between each *A. equuli* strain and other *Actinobacillus* species and the high divergence of the excluded *Actinobacillus* species, the *Actinobacillus* genus is represented by highly divergent species within and between different clades (Figure 1C and Additional file 1).

### Identification of haemolysis-associated genes within the *Actinobacillus* genus

**Figure 2:**
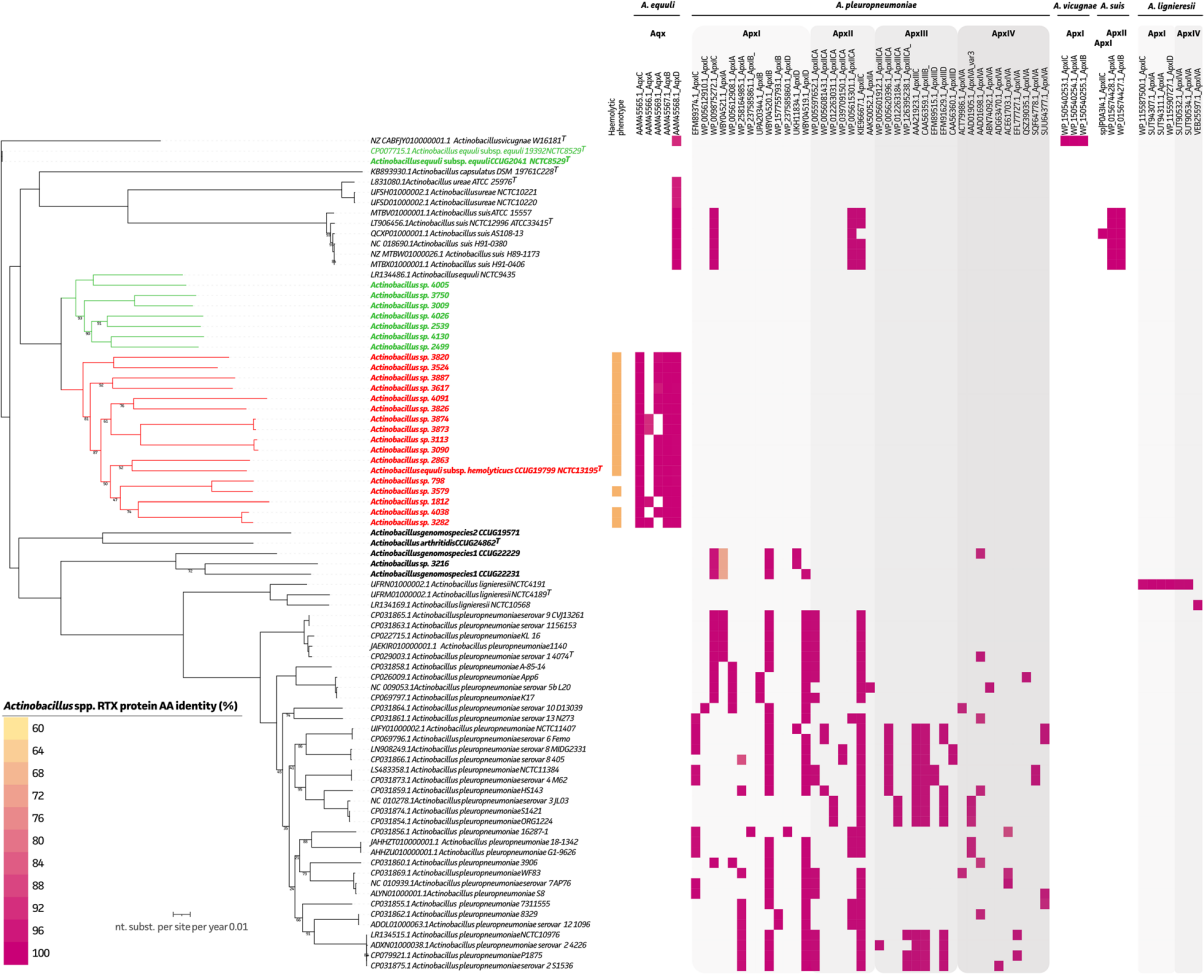
**Presence of haemolysis-associated genes within the genus of *Actinobacillus *sensu stricto.** An ML tree (1000 ultrafast bootstraps) highlighting phenotypic (orange) haemolysis of new *Actinobacillus* strains in relation to haemolysis-associated genes embedded within all *Actinobacillus *sensu stricto genomes, including RTX protein hits across the *Pasteurellaceae* family. Colour code (yellow-magenta) represents amino acid identity of the identified proteins as compared to the NCBI RTX protein hits. Only hits with amino acid similarity above 60% and 80% coverage are shown, and RTX-like proteins were excluded. A complete overview can be found in Additional file 4.

Based on the WGS data, all non-haemolytic isolates, and the *A. equuli* reference strains NCTC 9435 and NCTC 8529 lacked all four *A. equuli* specific RTX operon proteins (AqxCABD) (Figure 2; green). All other *A. equuli* isolates (*n* = 16) and the *A. equuli* subsp. *haemolyticus* reference strain CCUG 19799^T^ had a complete RTX operon (Aqx(CABD)) embedded within their genomes (Figure 2; red). Noteworthy, strain 798 and 1812 showed no clear haemolysis when studied on blood agar plates. Nevertheless, none of the RTX operon proteins were absent or showed deviating amino acid identities. Interestingly, the *aqxD* gene was identified in all *A. equuli* subps. *haemolyticus*, *A. suis*, *A. urea*, and *A. vicugnae* strains, belonging to the same phylogenetic clade. In the *A. vicugnae* strain also the ApxI(CAB) proteins were identified. In all *A. suis* strains, an incomplete ApxI(CAB) operon and partial ApxII(CA) operon was found (Figure 2).

While genes encoding the RTX proteins were widely detected in all *A. pleuropneumoniae* (ApxI, ApxII, ApxIII, and ApxIV operons or combinations), also *Actinobacillus* genomospecies 1 isolate 3216 and both *Actinobacillus* genomospecies 1 reference strains CCUG 22229 and CCUG 22231, showed the presence of the *A. pleuropneumoniae* associated ApxI(CABD) operon, with an ApxIA protein showing a mean 70.5% (± 0.1%) amino acid homology. For the *A. lignieresii* strain NCTC4191, an ApxI(CAD) operon was identified, which was not found in the NCTC4189^T^ strain. In some of the excluded *Actinobacillus* species, also RTX operon genes were identified. Interestingly, all tree *A. lignieresii* strains, showed the presence of an ApxIV protein. In “*A. porcitonsillarum*” 9953L55 and *A. minor* 202, a complete ApxI(CABD) operon was present. While these were absent in the NM305^T^ strain, this strains genome showed the presence of an LktB protein from *Mannheimia haemolytica* with 71% protein homology. In the case of *A. seminis* NCTC1051^T^ and *A. porcinus* NM319^T^, an ApxIIIA protein was detected within their genomes. In the former, *A. seminis* NCTC1051^T^, also conserved *Pasteurella aerogenes*-derived PaxB and PaxD proteins were identified. A complete overview of the RTX protein distribution within the *Actinobacillus* genus is given in Additional file 6.

### Antimicrobial susceptibility testing

The results of the antimicrobial susceptibility testing, including the results of the quality control strains, are presented in Table 1. The distribution of the MIC values of most antimicrobial agents showed a unimodal distribution in the *A. equuli* isolates, except for penicillin, ampicillin, and chloramphenicol. Additionally, MIC values for the (potentiated; i.e., strengthen the antimicrobial effect of sulphonamides with the addition of trimethoprim) sulphonamides were spread out over a very wide MIC range. This wide distribution may have caused the NRI method to possibly falsely assign part of the strains as having acquired resistance towards sulfisoxazole, while for the trimethoprim/sulfamethoxazole combination only a putative wild-type population could be described. For penicillin, ampicillin, and chloramphenicol, only isolate 3887 was identified as a non-wild-type isolate, using the ECOFF as determined by the NRI method. The MIC values of the *Actinobacillus* genomospecies 1 strain 3216 were within the wild-type ranges of the *A. equuli* strains and no genetic resistance determinants were observed using the WGS data, suggesting this isolate also did not acquire resistance to any veterinary relevant antimicrobial agent.

**Figure 3:**
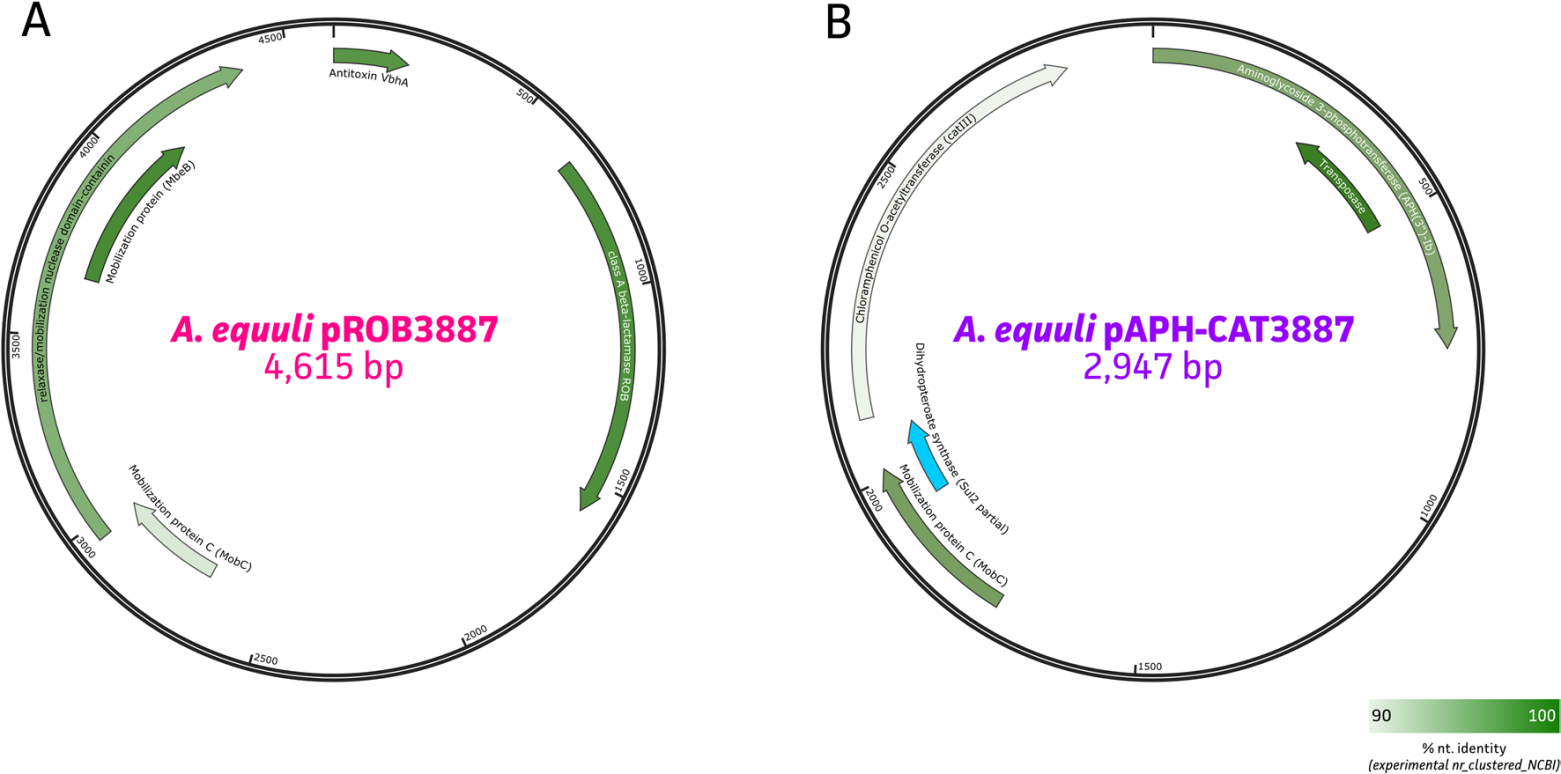
**Evaluation of AMR-associated genes within newly sequenced Actinobacillus spp. genomes.** **A** Annotation of the *A. equuli* pROB3887 plasmid carrying the class A beta-lactamase ROB-1; **B** Annotation of the *A. equuli* pAPH-CAT3887 plasmid carrying the aminoglycoside 3-phosphatase APH(3'')-Ib, chloramphenicol O-acetyltransferase *catIII*, and truncated dihydropteroate synthase *sul2* gene (blue).

Again, WGS data were used to evaluate the presence of antimicrobial resistance associated genes. Analysis of the sequencing data showed that all strains showed the presence of the global regulator *crp* gene within the genome. Genomic presence of the multidrug resistance transporter *msbA* gene was shown in 53% (16/30) of the clinical strains. Though, none of these could be correlated with observed phenotypic acquired resistance. Interestingly, the *A. equuli* subsp. *haemolyticus* strain 3887 showed the presence of two plasmids carrying various antimicrobial resistance genes. As shown in Figure 3, the class A beta-lactamase ROB-1, the aminoglycoside 3-phosphatase APH(3'')-Ib and chloramphenicol O-acetyltransferase *catIII* were identified on two different plasmids, named *A. equuli* pROB3887 (4615 bp) and pAPH-CAT3887 (2947 bp), respectively (Figures 3A and B). Further annotation of the latter showed the presence of a truncated dihydropteroate synthase *sul2* gene (17.7% coverage; Figure 3B; blue). In-depth characterization showed closest matches with the pB1000 plasmid from *Haemophilus influenzae* strain BB1053 (GU080065) and pMHSCS1 plasmid from *Mannheimia haemolytica* (AJ249249), respectively. While the pROB3887 plasmid was considered mobilizable, the pAPH-CAT3887 plasmid was classified as non-mobilizable, possibly due to the truncation of the MobA mobilization protein in the 2947 bp plasmid. To validate the presence/absence of this plasmid in any other strains, all other strains were screened for these plasmids using a targeted multiplex PCR, showing no detection in any other of the currently targeted strains.

## Discussion

The present study provides new insights in the genetic diversity and presence of RTX virulence genes of the *Actinobacillus* genus. While focussing on equine clinical strains, also novel phenotypic and genetic data on acquired resistance in *A. equuli* were obtained. In general, identification of *A. equuli* is considered difficult due to its close relationship to *A. suis* [32, 56]. The use of the 16S rRNA gene was compared to a new MLST and whole genome SNP, showing that 16S rRNA gene sequencing was indeed least reliable in differentiating *Actinobacillus* species, while whole genome SNP analysis performed best. The use of 16S rRNA gene sequencing was also shown to not provide sufficient resolution and lack of specificity in previous research on *Bacillus* and *Actinobacillus* species [56, 57]. Interestingly, the *Actinobacillus* genomospecies 1 clade could be properly distinguished from other *Actinobacillus* species using the 16S rRNA gene only. This was not the case for *A. suis* and *Actinobacillus* genomospecies 2 which clustered amongst *A. equuli* strains. Here a putative new MLST scheme was used based on previously published schemes for *Pasteurellaceae* [41, 45]. The inclusion of the *adk* (adenylate kinase), *atpG* (ATP FOF1 synthase subunit gamma), *deoD* (purine nucleoside phosphorylase), *zwf* (glucose-6-phosphate dehydrogenase), *recA* (recombinase A), *mdh* (malate dehydrogenase), and *pgi* (glucose-6-phosphate isomerase) genes allowed to distinguish *A. equuli* from other *Actinobacillus* species, though it did not support the distinction between *A. equuli* subsp. *equuli* and *A. equuli* subsp. *haemolyticus*. This was only possible when using the SNP-based phylogenetic inference [40]. Even though *A. equuli* and *A. suis* might be separated by detection of specific virulence genes (*apx*/*aqx*), sequencing certain housekeeping genes (e.g., *infB*), or by biochemical tests such as fermentation of D(-)-mannitol and cellobiose and β-glucosidase production, these tests are not always easy to interpret [58–60]. Over the years improvements to these tests have been made [61], still biochemical tests are progressively replaced by faster and easier techniques such as MALDI-TOF MS in diagnostic laboratories [33, 62]. MALDI-TOF MS, however, was not able to unequivocally identify all clinical strains as *Actinobacillus equuli* and did not support further subspecies classification. Albeit, both subspecies were included in initial versions of the Bruker Biotyper 3.0 database [62]. In the most recent version of the Bruker Biotyper database that was used in this study, the software highlights that the species *A. equuli*, *A. lignieresii*, *A. pleuropneumoniae*, and *A. suis* have highly similar 16S rRNA gene and MALDI-TOF MS spectra. Therefore, distinguishing these species remains difficult. Interestingly, one of the clinical strains (3216) could not be correctly identified at all using MALDI-TOF MS, but this is mainly because *Actinobacillus* genomospecies 1 is not represented in the Bruker database. In addition, using the rMLST analysis, the closest match for strain 3216 was *A. lignieresi* (60%). Hence, we supplemented current data with other *Actinobacillus* species, including *Actinobacillus* genomospecies 1, *Actinobacillus* genomospecies 2, and *A. arthritidis* for which reference/type strains were sequenced. To the authors knowledge these are the first whole genome sequences available for these species. This allowed to classify the 3216 strain to *Actinobacillus* genomospecies 1 as it showed closest phylogenetic relation to the two *Actinobacillus* genomospecies 1 type strains, based on 16S rRNA gene, a putative MLST scheme, and whole genome SNP phylogenetic inferences. Of note, both MALDI-TOF MS and pubMLST showed close matches to the *A. lignieresii* species, which seemed to be the closest related species within the SNP-based tree (Figure 1C). First genome sequences of *Actinobacillus* genomospecies 1, *Actinobacillus* genomospecies 2, and *A. arthritidis* represented distinct clades within the *Actinobacillus* genus. The ability to identify and classify these lesser-known bacterial species is important as they have been linked to arthritis, stomatitis, and septicemia in horses [3, 13, 33]. Even though sequencing represents an interesting tool, existing databases (e.g*.* rMLST on pubMLST) also exhibit limitations if no closely linked species are present. Hence, lowered sequencing costs and new methodologies will encourage the availability of more divergent bacterial species, even from unculturable isolates from metagenomics sequencing [63–65].

It is generally accepted that *A. suis* strains carry ApxI(CABD) and ApxII(CA) proteins, while *A. equuli* subsp. *haemolyticus* isolates carry the Aqx(CABD) operon encoding the Aqx toxin and *A. equuli* subsp. *equuli* do not carry any RTX member toxins [66–68]. Indeed, this was confirmed in our study, where a clear distinction between the *A. equuli* subsp. *haemolyticus* and *A. equuli* subsp. *equuli* could be made with the presence/absence of the complete Aqx(CABD) operon. For the *A. suis* strains, incomplete ApxI(CAB) and ApxII(CA), operons were identified. These are thought to be complemented by the presence of the Aqx(D) protein. Within the *A. pleuropneumoniae* population, various ApxIV variants were identified, which was limited to this species and its phylogenetically close relative, *A. liqnieresii*. Also, the AqxD protein was identified in all genomes of *A. equuli* subsp. *haemolyticus*, *A. suis*, *A. ureae*, and *A. vicugnae*. The wider distribution of the AqxD protein suggests a putative other functional role within these species. Indeed, RTX exoproteins represent a highly diverse family, with its most studied function linked to enzymatic cytotoxins in a wide variety of bacteria, including various members of the *Enterobacteriaceae* and *Pasteurellaceae*. In addition, RTX proteins have been described to be hydrolytic enzymes with protease/lipase activity (e.g., *Serratia* and *Pseudomonas*), bacteriocin activity and nodulation (e.g., *Rhizobium* and *Agrobacterium*), and motility in *Cyanobacteria* [69]. Though, its exact role(s) in diverse bacterial species, including *Actinobacillus* species, remains to be elucidated. This might also be the case for the identified LktB and PaxD/PaxB proteins in *A. minor* and *A. seminis*, respectively. As described before, a wide variety of RTX toxin clusters have been identified in different *Actinobacillus* species [70]. For *A. pleuropneumoniae* four different clusters (ApxI-IV) have been described [71]. Our data shows the occurrence of these four major RTX clusters associated with their genomic clades. While complete ApxI(CABD) and ApxIII(CABD) operons were identified in previously sequenced *A. pleuropneumoniae* strains, no complete ApxII(CABD) operons were found. Also, most *A. pleuropneumoniae* strains harbour one or more incomplete RTX operons. Even though RTX genes from *E. coli* and *B. pertussis* were shown to be complementary in vitro, the complementary character for *Actinobacillus* species should be further elucidated [69]. Interestingly, our newly sequenced *Actinobacillus* genomospecies 1 strains also showed the presence of a complete ApxI(CABD) operon. Though, substantial mutations at the protein level were observed in the ApxI(A) protein. The absence of any of the RTX operons in the *Actinobacillus* genomospecies 2 reference strain is supported by previous phenotypic observations in an *Actinobacillus* genomospecies 2 strain isolated in Japan [6]. Overall, we conclude that sequencing-based methods contribute to a better understanding of the complete toxin landscape. It provides information on the completeness and potential complementary nature of the RTX gene operons without the need of species-specific primer sets.

Considering there are no veterinary clinical breakpoints for *A. equuli* [72] and that the therapeutic result is also strongly dependent on the stage of infection, the lack of acquired resistance does not guarantee a successful therapy [73]. On the other hand, the presence of acquired resistance can indeed hamper the in vivo efficacy of the antimicrobial agent. However, only for penicillin, ampicillin, and chloramphenicol, a remarkable increase in MIC values of 10 times above the highest MIC values of isolates belonging to the wild-type population was observed for isolate 3887. For oxacillin, the MIC value of isolate 3887 was merely twice as high as the highest MIC value of the rest of the tested population. This is probably due to the plasmid-encoded *bla*_ROB-1_ resistance mechanism that was observed in this isolate, which is commonly found among *Pasteurellaceae* and induces primarily resistance towards beta-lactamase susceptible penicillins [18]. It was therefore concluded that, even though the current collection of isolates is relatively small, there was no acquired resistance towards veterinary relevant antimicrobial agents in current equine *A. equuli* population, except for one isolate exhibiting acquired resistance towards beta-lactamase sensitive penicillins and chloramphenicol. Even though the anamnesis data joining the resistant strain were limited and could not be traced, various treatment periods with penicillin were described, which might be in line with the acquired beta-lactam resistance as observed using both MIC testing and WGS. While the *bla*_ROB-1_ gene was identified on a plasmid, named pROB3887, resistance markers against aminoglycosides (*aph(3″)-Ib*), and chloramphenicol (*catIII*) were present on a different plasmid (pAPH-CAT3887). Both plasmids were previously described in different *Pasteurellaceae* genera, of which similar plasmids carried a *sul2* and aminoglycoside *aph(3″)-Ib* gene. These genes are often observed together in *Actinobacillus*, *Pasteurella*, and *Mannheimia* species [30]. Also the *bla*_ROB-1_ beta-lactamase gene was shown to be widely occurring on plasmids within the family of *Pasteurellaceae* and sometimes collocated with *sul2* and *aph(3″)-Ib* [74].

In conclusion, our data highlight the added value of long-read nanopore WGS on the identification, virulence gene typing, and antimicrobial resistance testing of equine *Actinobacillus equuli* isolates at the highest resolution. Next to the availability of new WGS data on *Actinobacillus* genomospecies 1, *Actinobacillus* genomospecies 2, and *A. arthritidis*, our data allowed to deliver in-depth characterization of the genomic landscape of RTX-associated genes within the *Actinobacillus* genus. Furthermore, we identified two *A. equuli* specific plasmids, carrying various AMR genes which contribute to acquired AMR and dissemination of resistance in the *Pasteurellaceae *family.

## Supplementary Information

**Additional file 1. Overview of used WGS data for** ***Actinobacillus*** **spp. strains, including accessions, genome QC, and excluded strains based on divergence.**

**Additional file 2. Overview of rMLST analysis results for all used WGS genomes. Whenever percentages do not add up to 100%, not all ribosomal gene sequences could be proper classified or identified in the used genome.**

**Additional file 3. Overview of available MLST schemes for some members of the** ***Pasteurellaceae*** **family.**

**Additional file 4. Overview of identified RTX and RTX toxin-related proteins from all used WGS genomes. Percentages represent protein homologies.**

**Additional file 5. Overview of used plasmid-specific primers.**

**Additional file 6. Presence of haemolysis-associated genes within the*Actinobacillus* genus**. An ML tree (1000 ultrafast bootstraps) highlighting phenotypic (orange) haemolysis of new *Actinobacillus* strains in relation to haemolysis-associated genes embedded within all available *Actinobacillus* sp. genomes, including RTX protein hits across the *Pasteurellaceae* family. Colour code (yellow-magenta) represents amino acid identity of the identified proteins as compared to the NCBI RTX protein hits. Only hits with amino acid similarity above 60% and 80% coverage are shown, and RTX-like proteins were excluded. A complete overview can be found in Additional file 4.

## Data Availability

All data generated or analysed during this study are included in this published article and its supplementary information files.
