# Supplementary figures and images for: Whole genome sequencing to study antimicrobial resistance and RTX virulence genes in equine Actinobacillus isolates
Source: Vet Res. 2023 Apr 5;54:33. doi: 10.1186/s13567-023-01160-2 (PMC10074821; doi:10.1186/s13567-023-01160-2)

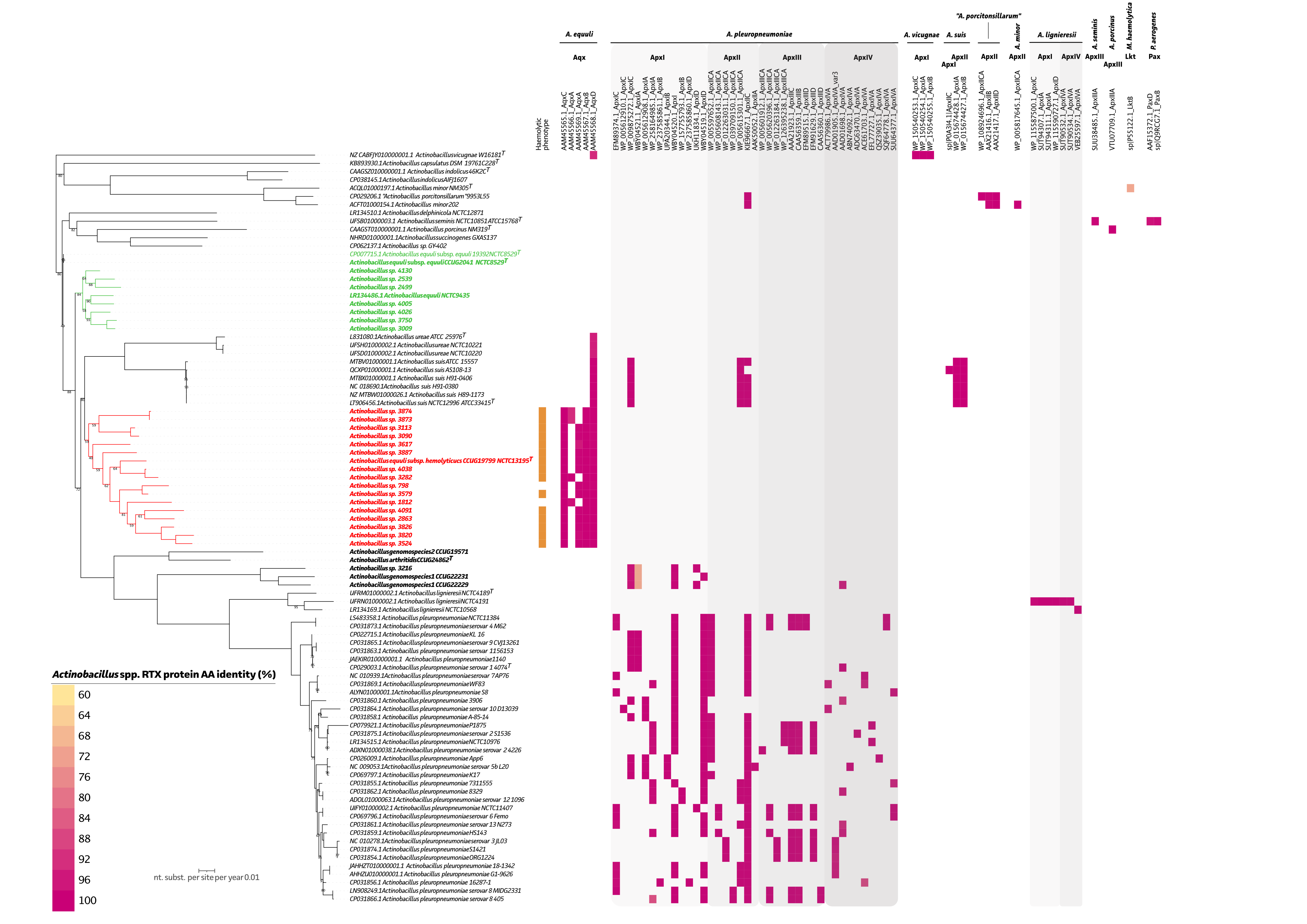

Supplement: Supplementary file 6 — Additional file 6. Presence of haemolysis-associated genes within theActinobacillus genus. An ML tree (1000 ultrafast bootstraps) highlighting phenotypic (orange) haemolysis of new Actinobacillus strains in relation to haemolysis-associated genes embedded within all available Actinobacillus sp. genomes, including RTX protein hits across the Pasteurellaceae family. Colour code (yellow-magenta) represents amino acid identity of the identified proteins as compared to the NCBI RTX protein hits. Only hits with amino acid similarity above 60% and 80% coverage are shown, and RTX-like proteins were excluded. A complete overview can be found in Additional file 4. [file 13567_2023_1160_MOESM6_ESM.tif]
